# Niosomal Nanocarriers for Enhanced Dermal Delivery of Epigallocatechin Gallate for Protection against Oxidative Stress of the Skin

**DOI:** 10.3390/pharmaceutics14040726

**Published:** 2022-03-28

**Authors:** Danhui Li, Nataly Martini, Zimei Wu, Shuo Chen, James Robert Falconer, Michelle Locke, Zhiwen Zhang, Jingyuan Wen

**Affiliations:** 1 School of Pharmacy, Faculty of Medical and Health Sciences, The University of Auckland, Auckland 1023, New Zealand; danhui.li@auckland.ac.nz (D.L.); n.martini@auckland.ac.nz (N.M.); z.wu@auckland.ac.nz (Z.W.); shuo.chen@auckland.ac.nz (S.C.); 2 Department of Plastic, School of Pharmacy, The University of Queensland, Pharmacy Australia Centre of Excellence, Brisbane, QLD 4102, Australia; j.falconer@uq.edu.au; 3 Reconstructive Surgery, Middlemore Hospital, Counties Manukau District Health Board, Auckland 2104, New Zealand; m.locke@auckland.ac.nz; 4 Shanghai Institute of Materia Medica, Chinese Academy of Sciences, Shanghai 201203, China; zwzhang0125@simm.ac.cn

**Keywords:** niosomes, catechin, dermal delivery, antioxidant activity, oxidative stress, skin barrier, penetration, cellular uptake

## Abstract

Among green tea catechins, epigallocatechin gallate (EGCG) is the most abundant and has the highest biological activities. This study aims to develop and statistically optimise an EGCG-loaded niosomal system to overcome the cutaneous barriers and provide an antioxidant effect. EGCG-niosomes were prepared by thin film hydration method and statistically optimised. The niosomes were characterised for size, zeta potential, morphology and entrapment efficiency. Ex vivo permeation and deposition studies were conducted using full-thickness human skin. Cell viability, lipid peroxidation, antioxidant enzyme activities after UVA-irradiation and cellular uptake were determined. The optimised niosomes were spherical and had a relatively uniform size of 235.4 ± 15.64 nm, with a zeta potential of −45.2 ± 0.03 mV and an EE of 53.05 ± 4.46%. The niosomes effectively prolonged drug release and demonstrated much greater skin penetration and deposition than free EGCG. They also increased cell survival after UVA-irradiation, reduced lipid peroxidation, and increased the antioxidant enzymes’ activities in human dermal fibroblasts (Fbs) compared to free EGCG. Finally, the uptake of niosomes was via energy-dependent endocytosis. The optimised niosomes have the potential to be used as a dermal carrier for antioxidants and other therapeutic compounds in the pharmaceutical and cosmetic industries.

## 1. Introduction

The skin is the largest organ of the human body, which makes it the direct target of oxidative stress due to the exposure to reactive oxygen species (ROS) from the surrounding environment. The most important function of human skin is protection by providing a barrier from pathogens, and physical and chemical damages. It also plays a crucial role in thermoregulation and endocrine function such as vitamin D synthesis [1,2]. The skin comprises three layers: the epidermis, which consists of keratinocytes; the dermis consisting of connective tissue, and the subcutaneous layer [3]. The epidermis can be divided into four layers, including the stratum corneum (SC), stratum granulosum, stratum spinosum and stratum basale. The dermis is composed of connective tissues, which are also rich in glands, white blood cells and blood vessels [4,5]. SC is the highly hydrophobic surface layer that contains 18 to 21 cell layers and is composed of corneocytes that are terminally differentiated keratinocytes anchored in a lipophilic matrix [3,4]. The ‘bricks and mortar’ model is often employed to describe the structure of SC, in which intercellular lipid accounts for 10% of the dry weight of this layer, and the rest is an intracellular protein (mainly keratin). Keratins are a family of alpha-helical polypeptides with a molecular weight ranging from 40,000 to 70,000 Daltons, making the corneocyte layers dense and relatively impervious to external compounds [6].

The skin is continuously exposed to environmental threats such as UV radiation, pollution, micro-organisms, and viruses, which lead to ROS production. ROS are also formed during the normal cellular metabolism and immune reactions. More than 80% of environmental ROS that damage the skin is produced by UV [7]. Antioxidants such as glutathione, ubiquinol and thiols inhibit oxidation reactions by donating electrons to free radicals [7]. Our bodies also produce enzymatic antioxidants, such as superoxide dismutase and glutathione peroxidases. Other non-enzymatic antioxidants such as vitamin E (alpha-tocopherol) and vitamin C (ascorbic acid) are obtained from the diet [8]. However, the antioxidants produced by our bodies are inadequate to protect against oxidative stress, and antioxidants are often used as dietary supplements to replenish the level of endogenous antioxidants and hence help to delay the onset of aging or diseases [8].

Catechins are a group of powerful antioxidants with health-promoting effects, and epigallocatechin gallate (EGCG) is one of the catechins found in green tea. EGCG has several beneficial effects on the skin, including anti-aging, anti-inflammatory, and anti-cancer properties. According to a study, treating normal human epidermal keratinocytes with EGCG prevented UVB-induced intracellular release of hydrogen peroxide while also inhibiting UVB-induced oxidative stress-mediated skin damage [9]. EGCG has been shown to inhibit UV-induced collagen production and collagenase transcription in human dermal fibroblasts [10]. In addition, on the human model, catechins were shown to have anti-aging functions [9]. A double-blind, placebo-controlled experiment of adult women found that catechins can reduce total sun damage when given as oral catechins supplements [11]. The oral administration route is generally the most accepted for drug administration, particularly for long-term prevention purposes. However, when administered orally, catechins readily undergo several metabolic transformations by intestinal microflora and enzymes; therefore, they are poorly bioavailable [12]. The application of EGCG is also limited by its unstable physiochemical properties, which can be degraded quickly. Many studies have reported that green tea catechins were vulnerable to degradation caused by the elevation of temperature, pH, and metal ions of incubation media [13]. The instability is part of the reason for the poor bioavailability and also presents as an issue in the manufacturing process. Therefore, the oral bioavailability of catechin represents a big challenge. Topical application of these bioactive compounds may be able to overcome the problem, as this route bypasses metabolism by the liver and gastrointestinal track with relatively low enzymatic degradation. However, the skin barrier, which is due to the SC layer, impedes the transport of exogenous compounds into the skin and restricts diffusion of external substances into the deeper dermis layer. Therefore, we hypothesise that loading EGCG into a drug carrier would help overcome the skin barrier, improve penetration into the deeper skin layers and improve their stability. The compound can therefore exert its beneficial effect at the site of interest.

Niosomes are versatile drug carrier systems that have been administered via various routes; they are surfactant-based nanocarriers that are mainly composed of non-ionic surfactant and cholesterol [14,15,16,17]. Niosomes have been extensively studied in topical drug delivery due to their ability to significantly improve penetration across the skin barrier and deposition in the dermis layer [18,19,20,21,22,23,24]. Drugs with a variety of physicochemical properties have been investigated for topical and transdermal delivery using niosomes, for example, diacerein [25], itraconazole [26], tretinoin [27], salidroside [28] and finasteride [29], demonstrating their advantages in topical delivery. In addition, a range of bioactive compounds has also been loaded into niosomes, such as curcumin, rutin, and Ginkgo biloba extract [30,31,32]. Silymarin-loaded niosomes demonstrated superior antioxidant activity over silymarin suspension in vitro [33].

In formulation development, various factors might impact the final product’s performance. Design of Experiment (DOE) is a planned set-up of experiments to acquire information efficiently and precisely. It applies to any process that has quantifiable inputs and outputs. DOE was first designed for agricultural applications, but it has since become a frequently used technique in process sectors, including the chemical, food and pharmaceutical industries [34]. It may be used to explore the effect of multiple variables on responses by altering them all at once in a small number of tests. By this strategy, the costs and time involved with the research and production of medicine may be significantly decreased [34,35]. Furthermore, it aids in the creation of the “best possible” formulation composition and gives a comprehensive knowledge of the process and product behaviors [36]. This study aimed to develop an optimal niosome formulation using the DOE methodology and evaluate the formulation for topical administration of EGCG for protecting the skin from external oxidative stress. An ex vivo investigation on human skin was carried out to evaluate drug deposition and the antioxidant activity of the EGCG-niosomes. The uptake of niosomes by human skin fibroblasts was also investigated.

## 2. Materials and Methods

### 2.1. Materials

Span^®^ 60, EGCG ≥ 98% (HPLC), cholesterol (CH), Triton™ X-100, dihexadecyl phosphate (DCP), Fluorescein 5(6)-isothiocyanate (FITC), Sulforhodamine B (SRB), trichloroacetic acid (TCA), dimethyl sulphoxide (DMSO), methanol and acetonitrile (ACN) were purchased from Merck (Merck, Kenilworth, NJ, USA). Dulbecco’s Modified Eagle Medium (DMEM) with high glucose and l-glutamine, Phosphate-Buffered Saline (PBS), Hank’s Balanced Salt Solution (HBSS), penicillin-streptomycin, fetal bovine serum of New Zealand origin (FBS), trypsin-EDTA, DAPI and CellTracker were purchased from Thermo Fisher Scientific (Auckland, New Zealand). Malondialdehyde (MDA), glutathione peroxidase (GSH-px) and superoxide dismutase (SOD) kits were purchased from Biovision (Biovision Inc., Milpitas, CA, USA). Trifluoroacetic acid (TFA) was purchased from Fluka (Fluka, Darmstadt, Germany). Distilled, deionised water was used throughout and was obtained from a Millipore water purifier. 

### 2.2. High-Pressure Liquid Chromatography Method for Quantification of EGCG

An Angilent Technologies 1100 series high pressure liquid chromatography (HPLC) system equipped with a vacuum degasser, autosampler, thermostatted column compartment and photodiode-array detector (PDA) was used. A C18 column (Jupiter, 250 × 4.6 mm, 5 mm, Phenomenex, Torrance, CA, USA) was used for HPLC method development for EGCG. The mobile phase consisted of Milli Q water (0.1% TFA) and methanol at 75:25 ratio. EGCG was analysed at flow rate of 0.8 mL/min, with an injection volume of 20 µL and wavelength of 280 nm at 25 °C. 

### 2.3. Preparation of EGCG Loaded Niosomes

A total of 150 mmol of surfactant, cholesterol and DCP (2 µmol) was dissolved in organic solvents (methanol/chloroform, 4:1, *v*/*v*) and then the mixture rotatory evaporated to form a thin lipid film on the wall at 45 °C. The the lipid film was purged with nitrogen to remove any organic solvents. The thin film was then hydrated with PBS (pH 7.4, 10% ethanol) containing 2 mg of EGCG at 58 °C to form EGCG-niosomes. The niosome suspension was then extruded through a 400 nm polyester membrane with an ER-1 extruder (Eastern Scientific, Rockville, MD, USA) for 10 cycles and then stored at 20–25 °C for the niosome membrane to anneal. 

### 2.4. Optimisation of Formulation with Design of Experiment

#### 2.4.1. Formulation Optimisation by 2^6−2^ Fractional Factorial Design

Based on the preliminary experiments and literature study, six independent variables (factors), namely surfactant type (X_1_), drug amount (X_2_), molar ratio of CH to surfactant (X_3_), DCP amount (X_4_), hydration medium volume (X_5_) and hydration time (X_6_) were selected to be evaluated for their effect on drug entrapment efficiency (EE), which was the dependable variable (response). The six factors were examined on two levels: low and high, which were represented by transform codes of -1 and +1, respectively. A one-quarter two-level six-factor (2^6−2^) fractional factorial design comprising 16 runs as highlighted in the design display table was constructed by Design-Expert^®^ 7.0 (Stat-Ease, Minneapolis, MN, USA). The factors and levels employed in the design are listed in Table 1. In order to estimate the experimental error and check the response curvature, duplicates were added at two centre points (one for each surfactant type), totally giving 20 runs. The batches were produced in random order. Data analysis was performed by using Design-Expert^®^ 7.0 statistical software. The main effect of variables and interactions were determined according to the Equation (1) listed below:

(1) $$E_{X}=\bar{y_{\left(+1\right)}}-\bar{y_{\left(-1\right)}}$$

Contribution was used to determine which factors were larger contributors than others and it is calculated as:

$${Contribution}_{x_{i}}\left(\%\right)=\frac{SS_{xi}}{SS_{total}}\times 100$$

where $SS_{xi}$ is the sum of square of factor $X_{i}$; $SS_{total}$ is the total sum of square. The data was tested for significance by analysis of variance (ANOVA) with a level of significance of 5% (*p* = 0.05).

#### 2.4.2. Optimisation of Entrapment Efficiency by Central Composite Design (CCD)

The key variables that were identified to have significant effects on the EE were subjected to the optimisation step. A CCD was used to determine the optimum conditions and to investigate how sensitive the response was to the changes in the settings of the independent variables. The CCD, as described previously, consists of a full factorial design with centre and star points, which generates enough information to fit a second-order polynomial model. The two influential factors, namely drug amount (X_1_) and ratio of CH to surfactant (X_2_) were chosen as independent variables and EE was assessed as dependent variable, the other factors were fixed based on the findings obtained from the screening design. A total of 13 experiments were performed, including five replicates on the centre point which improved the assessment of the response surface curvature and simplified the estimation of the model error. The levels of the factors in EGCG-niosome optimisation are shown in Table 2.

Data analysis was performed by using Design-Expert^®^ 7.0 statistical software. The data were tested for significance by analysis of variance (ANOVA) with a level of significance of 5% (*p* = 0.05). The second-order Equation (2) generated is described as:

(2) $$y=\beta_{o}+\beta_{1}X_{1}+\beta_{2}X_{2}+\beta_{11}X_{1}^{2}+\beta_{22}X_{2}^{2}+\beta_{12}X_{1}X_{2}$$

where *y* stands for the predicted response (dependent variable), $\beta_{o}$ is the intercept; $\beta_{1}-\beta_{22}$ are the regression coefficients; $X_{1}$ and $X_{2}$ stand for the main effect of the two factors; $X_{1}X_{2}$ is the interactions between the main effects; and $X_{1}^{2}$ $X_{2}^{2}$ are quadratic terms of the independent variables that are used to simulate the curvature of the designed space.

Checkpoint analyses were carried out to establish the reliability of the CCD regression model in describing composition parameters’ effect on entrapment efficiency. The optimum point was chosen according to the prediction based on the second-order equation. Predicted and experimental values were compared to determine the correlation extent between the actual and predicted responses.

### 2.5. Characterisation Studies

#### 2.5.1. Particle Size, Size Distribution and Zeta Potential Analysis

The mean particle size and polydispersity index (PDI) of the optimised EGCG niosome was determined by dynamic light scattering using the photon correlation spectroscopy (PCS) technique using a Zetasizer (Malvern instruments, Malvern, UK). A dilute suspension of the niosomes was prepared with Milli Q water. The size measurement was performed in triplicate at 25 °C.

The zeta potential (ζ), is an indicator of particle surface charge, which may arise from the adsorption of a charged species and/or from ionization of groups that at the surface of the formed particles. It determines particles’ stability in dispersion. To measure zeta potential of the optimised EGCG niosome, they were dispersed into Milli Q water (pH 7) and measured in triplicate using the Malvern Zetasizer.

#### 2.5.2. Entrapment Efficiency (EE%)

To separate the free and entrapped drugs, ultracentrifugation was used. In summary, the niosomal dispersion was centrifuged for 1 h at 4 °C at 41,000 rpm using a WX80 centrifuge (Beckman Sorvall, Waltham, MA, USA). The amount of EGCG in the supernatant was measured with HPLC (Agilent LC1100, Santa Clara, CA, USA) after particle separation by centrifugation. Then the niosome pellets were gently rinsed with PBS and then dissolved in a methanol solution containing 10% Triton™X-100 solution and then sonicated in a water bath sonicator for 10 min. The resulting liquid was filtered and then subjected to concentration determination by HPLC. The following Equation (3) was used to calculate the entrapment efficiency:

(3) $$Entrapment\;efficiency=\frac{\left(Total\;drug-Free\;drug\right)\;}{Total\;drug\;added}\times 100\%$$

#### 2.5.3. Morphology by Scanning Electron Microscopy (SEM)

SEM (XL30S FEG, Philips, Eindhoven, Netherlands) was used to study the optimised niosomes’ morphology. The niosome dispersion was diluted 20 times with Milli Q water before being dried on the grid. Gold and palladium sputter coating was applied before morphological evaluation at 25 kV.

#### 2.5.4. Differential Scanning Calorimetry (DSC) and Fourier Transform Infra-Red Spectroscopy (FTIR)

IR spectroscopy and DSC were used to investigate the drug’s interaction and entrapment in the vesicular structure. DSC (TA Instruments, New Castle, DE, USA, Q2000+ RCS40) was used to analyse the thermal properties of the optimised niosomes. Span60^®^, cholesterol, pure drug, and a physical mixture of these components and the lyophilised niosomes were placed into T-zero aluminum pans and hermetically sealed. The temperature rises to 200 °C at a rate of 10 °C/min from a starting temperature of 20 °C for all experimental runs.

FTIR spectra of the individual and mixture of formulation components and lyophilized niosomes were determined using a Bruker FTIP tensor 37 (Bruker Optics, Billerica, MA, USA) at a 4 cm^−1^ resolution between 500 and 4000 cm^−1^.

### 2.6. In Vitro Drug Release

In vitro release of drug-loaded EGCG-niosomes was studied using a Franz diffusion apparatus (FDC-6, Logan Instrument Corp, Somerset, NJ, USA). EGCG solution containing the equivalent quantity of EGCG as the drug loaded niosomes was used as a control. EGCG-niosomes and drug solution were added to the donor compartment of the Franz diffusion cell, with a cellulose membrane (MW 12,000–14,000, Membra-Cel ^®^, Viskase, Lombard, IL, USA) sandwiched between the donor and receptor chambers. The receptor chamber was filled with PBS (pH 5.5) and the temperature was maintained at 37 ± 1 °C. Aliquots (400 μL) were withdrawn at pre-determined time points (15 min, 30 min, 1 h, 2 h, 3 h, 4 h, 6 h, 8 h, 12 h and 24 h) and replaced with fresh PBS (400 μL). The samples were centrifuged at 13,000 rpm for 30 min, and the supernatant was filtered (0.22 μm) and analysed with the HPLC method.

To determine the release mechanism, the release data were fitted into the Korsmeyer–Peppas model:

$$Q_{t}=K_{k}t^{n}$$

where *Q_t_* is the cumulative drug released at time *t*, *k_k_* is a kinetic constant characteristic of the drug/polymer system, and *n* is an exponent describing the release mechanism.

### 2.7. Ex Vivo Skin Permeation and Deposition Studies

#### 2.7.1. Skin Permeation Studies

The full-thickness skin samples were kindly donated by patients who underwent elective skin reduction surgeries at Middlemore hospital, Auckland. This project has been approved by the University of Auckland’s Human Participants Ethics Committee (approval number: 010990). The skin samples were stored at −20 °C immediately after the surgeries and used within one month.

The ex vivo skin permeation and deposition studies were carried out using Franz diffusion apparatus (FDC-6, Logan Instrument Corp, Somerset, NJ, USA). EGCG-niosomes and drug solution were added to the donor compartment of the Franz diffusion cell, with a piece of full-thickness human skin sandwiched between the donor and receptor chambers (effective diffusional area: 1.77 cm^2^). The receptor chambers were filled with PBS (pH 5.5) and the temperature was maintained at 37 ± 1 °C. Prior to the experiments, the integrity of the skin samples was verified by a Millicell-ERS equipment (Millipore, Burlington, MA, USA) to determine the electrical resistance (ER) across the skin. The skin samples that had an ER value above the cut-off value of 27.4 kΩ·cm^2^ were used in the study and equilibrated in PBS for 4 h before the study. For the permeation test, EGCG-niosome suspension and EGCG solution (containing an equivalent amount of EGCG as the drug loaded niosomes) were added to the donor compartments. Aliquots (400 μL) were withdrawn at 12 h and 24 h and replaced with fresh PBS (400 μL). The samples were centrifuged at 13,000 rpm for 30 min, and the supernatant was filtered (0.22 μm) and the drug concentration was determined by HPLC.

#### 2.7.2. Drug Deposition in the Skin

The skin tissues were removed from the Franz diffusion cells after 12 h and 24 h of the deposition study, and the surface of the skin tissue was thoroughly cleaned with methanol and then placed on a tissue cutting board. The SC layer of the skin was removed by a tape-stripping method with Scotch^®^ Magic tapes (3M, Maplewood, MN, USA) [37]. A tape and a 2 kg weight were placed on each skin sample for 10 s, then peeled with forceps, and 15 strippings were applied consecutively to remove the SC. Subsequently, the skin was cut into smaller pieces and 60 mg of skin tissue was placed in a MACS™ tube (Mitenyi Biotec Inc, Cambridge, MA, USA) with methanol, then dissociated using a dissociator (Mitenyi Biotec Inc, Cambridge, MA, USA). Protein was precipitated by adding TCA, then centrifuged at 13,000 rpm for 30 min, and the supernatants were filtered and analysed by HPLC.

#### 2.7.3. Visualisation of Skin Penetration and Deposition

FITC was added to the hydration medium to prepare FITC-labelled niosomes. Full-thickness human skin was placed between the donor and the receptor chamber of the Franz cells, and FITC labelled niosomes were added to the donor compartment. The skin samples were removed after 12 h and thoroughly cleaned. They were frozen and directly embedded in wax and then cut into sections with a microtome. The tissue sections were fixed on glass slides and then observed using a fluorescence microscope (DMIL LED, Leica, Wetzlar, Germany), and the images were captured.

### 2.8. Antioxidant Effect of EGCG Loaded Niosomes on Human Fibroblasts

#### 2.8.1. Cell Culture

The primary human fibroblasts (Fbs) were obtained from the American Type Culture Collection (ATCC, Manassas, VA, USA). Cells were routinely maintained in complete DMEM medium in T-75 tissue culture flasks (Corning, Phoenix, AZ, USA) at 37 °C in an atmosphere of 5% CO^2^ and 95% relative humidity. Complete DMEM medium was prepared by adding 10% fetal bovine serum, 1% penicillin-streptomycin-glutamine, and 1% nonessential amino acids. Culture medium was changed every 2 days until cells grew to 90% confluence. 

#### 2.8.2. Cellular Viability after UVA-Irradiation Using Sulforhodamine B (SRB) Assay

Optimised EGCG-loaded niosomes were prepared and centrifuged as per the above-mentioned method and were resuspended in cell culture medium. Fbs were seeded in 96-well plates (5000 cells/well) 24 h before UVA-irradiation to allow cells to attach. To administer UVA-irradiation, a UVA lamp (EN-160 L/FE, Spectroline, Melville, NY, USA) with a dose of 0.72 J/cm^2^ and wavelength of 320–400nm and wave peak at 365 nm was used. After irradiation, EGCG niosome suspension and free EGCG in culture medium solution were added to the 96-well and incubated for 24 h. The SRB assay was used to assess the cell viability. Briefly, the cells were gently washed with ice-cold PBS and fixed with 10% TCA, then 0.1 mL of 0.4% (*w*/*v*) SRB in acetic acid was added to stain the cellular proteins. The cell-bound dye was extracted using 0.1 mL 10 mM unbuffered Tris base solution (pH 10.5), and absorbance was measured at 596 nm with a plate reader (SpectraMax^®^ Plus, Molecular Devices, San Jose, CA, USA). Cell viability was expressed as a percentage of the control.

#### 2.8.3. Intracellular MDA Level and the Antioxidant Enzyme Activities

EGCG and EGCG niosomes were diluted with a serum-free medium and then added to the cells after being irradiated by UV light and then cultured for 24 h. After incubation, cells were removed from the 6-well plate and the amount of MDA was determined by the MDA assay kits. The antioxidant enzyme activities of SOD and GSH-px were determined using the same method described above, and the cellular enzymatic activities were determined using the respective assay kits.

### 2.9. Cellular Uptake of Niosomes by Human Fibroblasts

FITC was added to the hydration medium to prepare FITC-labelled niosomes, then subjected to centrifugation to remove free FITC. The niosome pellets were then resuspended in the medium and diluted to the predetermined concentrations. The control solution was prepared by dissolving FITC in DMSO and then diluted with the medium. For the uptake studies, Fbs suspension (5 ×10^5^ cell in 5 mL) was seeded onto Petri dishes (100 mm, Corning, Phoenix, AZ, USA), fed with completed DMEM every 2 days and incubated at 37 °C in an atmosphere of 5% CO2 and 95% relative humidity to allow cells to attach and proliferate. On reaching 90% confluence, the culture medium was replaced with 2 mL of HBSS. After incubation at 37 °C for 15 min, the HBSS was replaced with FTIC-labelled niosomes at concentrations from 50 to 2000 µg/mL to determine the effect of concentration on cellular uptake; to study the effected of incubation temperature and duration, FITC-labelled niosomes were incubated with Fbs at 4 and 37 °C for 0.5–24 h and at 37 °C for 0.5–24 h, respectively. Then the cells were washed with ice-cold HBSS for three times and then the cells were collected into a tube containing lysis medium (Methanol with 10% Triton™ X-100 solution), followed by ultrasonication for 15 min. Finally, 25 µL of the cell lysates was subjected to BCA protein assay using a Pierce^®^ BCA protein assay kit (Thermo Fisher Scientific, Waltham, MA, USA) to determine the amount of protein in the cells. The remainder of the cell lysates were subjected for quantitative measurement using a fluorescein spectrophotometer (PerkinElmer Precisely, Waltham, MA, USA), at excitation wavelength of 495 nm, emission wavelength of 525 nm.

A Confocal Laser Scanning Microscope (CLSM) was used to examine whether niosomes were taken up and localised intracellularly or were simply adsorbed onto the cell surface. Fbs were transferred into 2-well chamber slides (BD Falcon, Phoenix, AZ, USA) at a density of 2 × 10^5^ cells/well (5 × 10^4^ cells/cm^2^) and grown in complete DMEM culture medium. The cells were treated with FITC-labeled niosome (2 mL) at the given doses for 2 h at 37 °C. After incubation, the cells were washed with ice-cold HBSS and then incubated with serum free medium for 15 min. Then the medium was withdrawn and a cytoplasm dye (CellTracker, Invitrogen, Auckland, New Zealand) (5 µM) in serum-free medium for 30 min in a cell incubator at 37 °C. After incubation, the cells were washed with PBS followed by a fixation solution of 3% paraformaldehyde for 30 min at room temperature. Then nuclei staining dye, DAPI (100 nM) (Invitrogen, Auckland, New Zealand) was added to the cells for 3 min. The culture chambers were removed, and the slides were rinsed and mounted with CITI-Fluor (Electron Microscopy Sciences, Hatfield, PA, USA). Coverslips were cemented in place with application of nail polish around their edges. Then the slides were observed by a confocal microscope FV1000 (Olympus, Hamburg, Germany).

### 2.10. Statistical Analysis

Statistical analysis was performed using the GraphPad Prism^®^ (GraphPad, San Diego, CA, USA) version 8.0 software via one-way ANOVA. A *p*-value of < 0.05 was considered the minimum level of significance. All data were expressed as mean ± SD.

## 3. Results

### 3.1. Formulation Development and Optimisation

Six formulation variables: surfactant type (X_1_), drug amount (X_2_), molar ratio of CH to surfactant (X_3_) and DCP (X_4_), hydration medium amount (X_5_) and hydration time (X_6_) were selected, and their effect on drug entrapment efficiency (Y) was evaluated. The experimental matrix for EGCG-niosome and responses of different batches obtained are presented in Table 3. The EE of EGCG in the niosomes had a range from 2.3 to 49%, suggesting that the factors investigated were influential on the drug encapsulation. Calculations were carried out based on the responses to determine the main effects of the factors and the interaction effects 

The results calculated to determine the main effects of the factors and the interaction effect are shown in Table 4.

After the estimation of the main effects, ANOVA was performed to determine the significant factors. The ANOVA results of the EGCG-niosome are shown in Table 5. A *p* value less than 0.05 (*p* < 0.05) indicated the effect was statistically significant. In this screening, surfactant type (X_1_), drug amount (X_2_), and the ratio of CH to surfactant (X_3_) were significantly influential on the response. The ANOVA showed that none of the two-factor interactions had a significant effect in the EGCG-niosome screening experiment. Surfactant type (X_1_), drug amount (X_2_) and the ratio of CH to surfactant (X_3_) were significantly influential factors on EE. Surfactant type (X_1_) played an important role in determining EE, and therefore, in the optimisation step, Span 60 was used, whereas drug amount (X_2_) and the ratio of CH to surfactant (X_3_) were optimised.

### 3.2. Optimisation of EE by CCD

In this step, significant factors detected by the screening design were optimised using a CCD. This design provides a solid foundation for generating a response surface plot, from which it is possible to get a target response. In the current study, it was the maximum EE% that the optimisation aimed to achieve. Transformed values of all the batches along with results of EGCG-niosome are shown in Table 6.

Equation (4) below represents the polynomial model for EGCG-niosome as obtained from the above experiment.

(4) $$Y\left(\mathrm{EE}\%\right)=47.06-3.98X_{2}+2.61X_{3}-4.30X_{2}^{2}-2.14X_{3}^{2}-0.61X_{2}X_{3}$$

**Figure 1:**
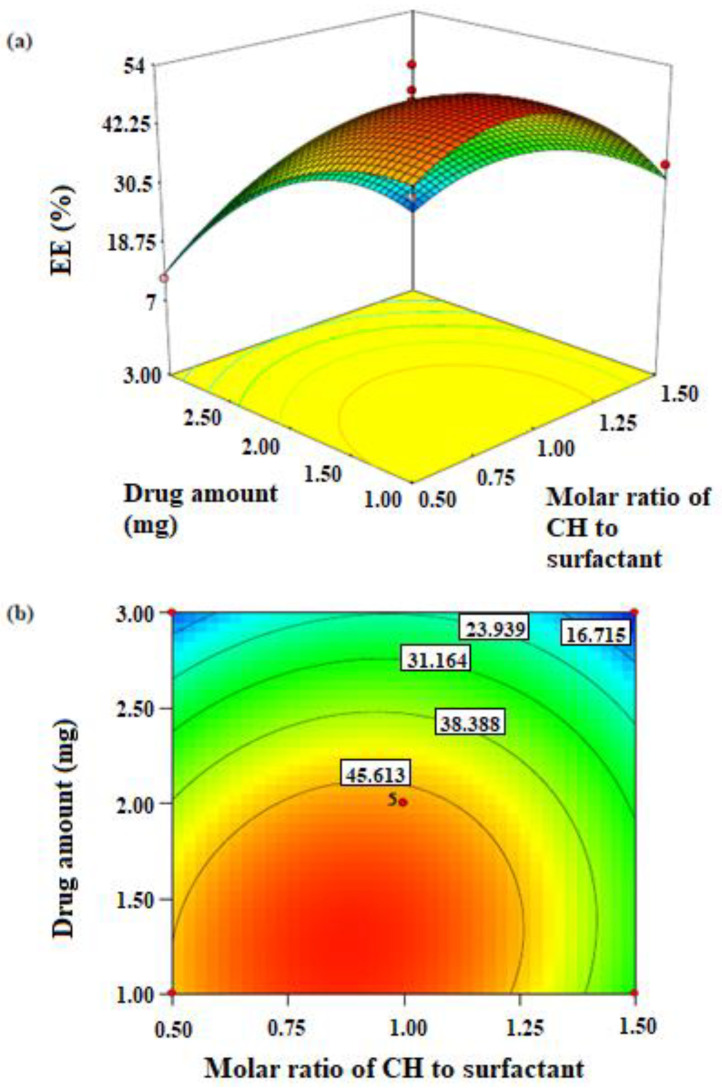
(**a**) Three-dimensional surface plot for EE of EGCG-niosome as a function of the formulation variables. (**b**) Contour plot for EE of EGCG-niosome as a function of the formulation variables.

The correlation coefficient ($r^{2}$) of 0.92 indicated that the model fitted the data very well and the ANOVA of the model reported a high significance (*p* < 0.001) (Table 7). The three-dimensional response surface and contour plots showing the variation in the entrapment efficiency with changes in drug amount (X_2_) and CH to surfactant ratio (X_3_) are presented in Figure 1. The highest EE was predicted to be achieved when drug amount (X_2_) is 1.4 mg and the molar ratio of CH to surfactant (X_3_) is 0.9.

### 3.3. Check Point Analysis

Having studied the effect of independent variables on the response, EE%, the levels of the factors were further determined by the optimisation process. Check points were evaluated to confirm the mathematic models’ predictivity by comparing the experimental EE (mean value out of four experiments) with the predicted value. In EGCG-niosome, the average experimental EE was 53.05 ± 4.46%, which was close to the predicted value EE of 53% with low percentage of bias (0.4%), suggesting that the optimised formulation parameters were reliable. The optimised formulation composition for EGCG-niosome is shown in Table 8, and the following characterisation studies were carried out on the optimised EGCG-niosome.

### 3.4. Characterisation of EGCG-Loaded Niosomes

The developed HPLC method was validated for linearity, repeatability, accuracy and sensitivity as per International Conference on Harmonisation (ICH) Q2(R1) guidelines. The standard curve was linear in the range between 1.93 to 145 µg/mL with a correlation coefficient (*r^2^*) of 0.999. Percentage of coefficient of variation (% CV) was determined to assess instrumental precision; both instrumental precision and intra-assay precision had % CV of less than 1.5%, indicating the method for EGCG is precise. Intermediate precision of the method was determined by assessing intra-day and inter-day repeatability; the % CV values were below 2.5%, which is acceptable according to the ICH guidelines. The sensitivity of the method was determined by limit of detection (LOD) and limit of quantification (LOQ), which were 0.33 µg/mL and 0.98 µg/mL, respectively.

### 3.5. Particle Size, Size Distribution, Zeta Potential Analysis and EE%

The average particle size of optimised EGCG-niosomes was 235.4 ± 15.64 nm, and the PDI value was 0.267 ± 0.053. A PDI of less than 0.5 indicates a narrow distribution of the particles [17]. EGCG-niosomes had a zeta potential of −45.2 ± 0.03 mV and EE% of 53.05 ± 4.46%.

### 3.6. Morphological Study

**Figure 2:**
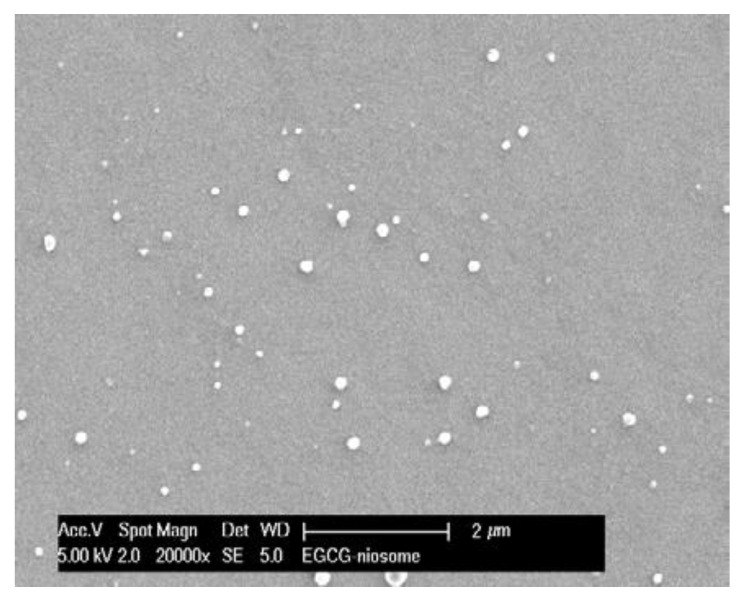
Scanning Electron Microscopy (SEM) image of the optimised EGCG niosomes.

As shown in Figure 2, the niosomes were 200 to 300 nm, spherical in shape with a closed vesicular structure and narrow size distribution. These findings were consistent with the size determined by the Zetasizer.

### 3.7. DSC and FTIR

**Figure 3:**
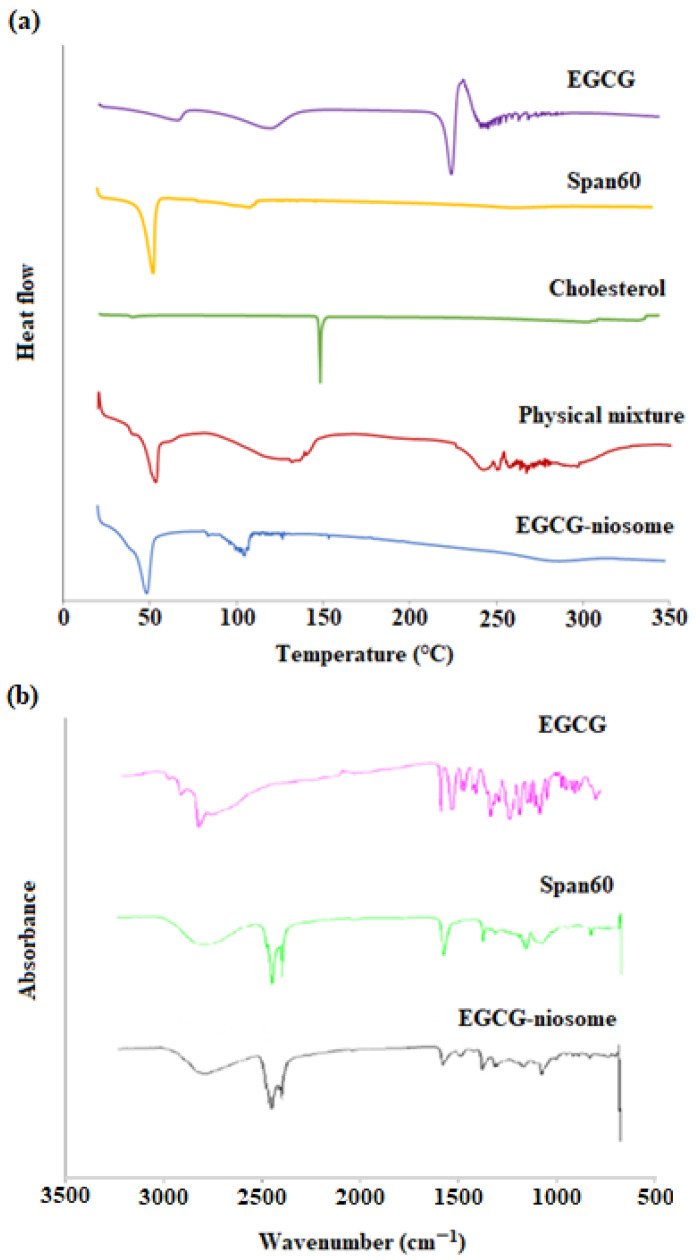
(**a**) Differential Scanning Calorimetry (DSC) thermograms and (**b**) Fourier Transform Infra-red Spectroscopy (FTIR) spectra of EGCG, Span 60 and EGCG-niosomes.

The DSC curves of the optimised EGCG niosomes, physical mixture of the formulation components, cholesterol, surfactant and EGCG are shown in Figure 3a. The endothermic peaks for Span 60 and cholesterol were 53 °C and 149 °C, respectively, which correspond to their melting points. The endothermic transition of EGCG (120 and 225 °C) are also reported in other studies. The physical mixture of formulation components showed similar transitions as EGCG and surfactant, where the characteristic peaks were not observed with EGCG niosomes. Additional peaks were found in EGCG niosomes between 100 to 150 °C, indicating there were interactions between the excipients. A large peak that appeared between 200 and 300 °C in EGCG-niosomes may suggest drug and excipient breakdown. FTIR spectroscopy verified the above results (Figure 3b. The FTIR graph showed the characteristic peaks for EGCG such –C–O stretching at 1200–1000 cm^−1^ and –C=C stretching at 1600–1500 cm^−1^. The spectrum of EGCG niosomes was similar to the surfactant; the other characteristic peaks were not observed, which confirmed the encapsulation of EGCG.

### 3.8. In Vitro Drug Release Profile

**Figure 4:**
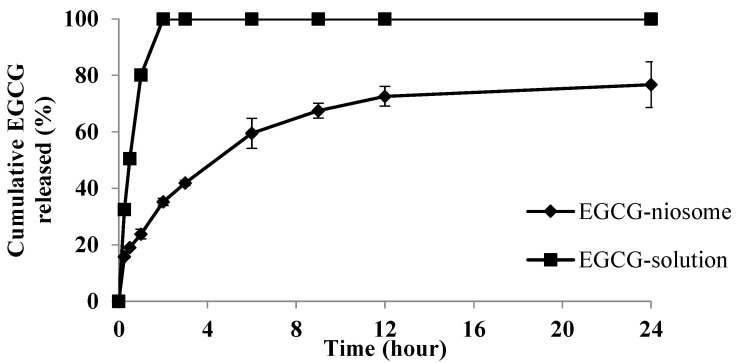
In vitro drug release of EGCG-niosomes and EGCG solution (mean ± SD, *n* = 3).

The in vitro drug release of EGCG from niosomes was examined using Franz diffusion cells. Figure 4 shows the release profile for control (EGCG solution) and EGCG-niosomes. Within 2 h, the EGCG solution was released immediately. The EGCG-niosomes, on the other hand, displayed a biphasic phase; around 35% of EGCG were released from the niosomes within the first 3 h, and then a sustained release was observed over 21 h, with 73% of EGCG was release at the end of the study. As shown in Table 9, the release data was fitted in several mathematical models of release kinetics. Based on the results, EGCG release from niosomes followed the Korsmeyer–Peppas model (*r*^2^ = 0.996). The release exponents were found to be 0.461, which indicates the drug release was governed by an anomalous diffusion mechanism with multiple steps.

### 3.9. Ex Vivo Skin Permeation and Deposition Studies

**Figure 5:**
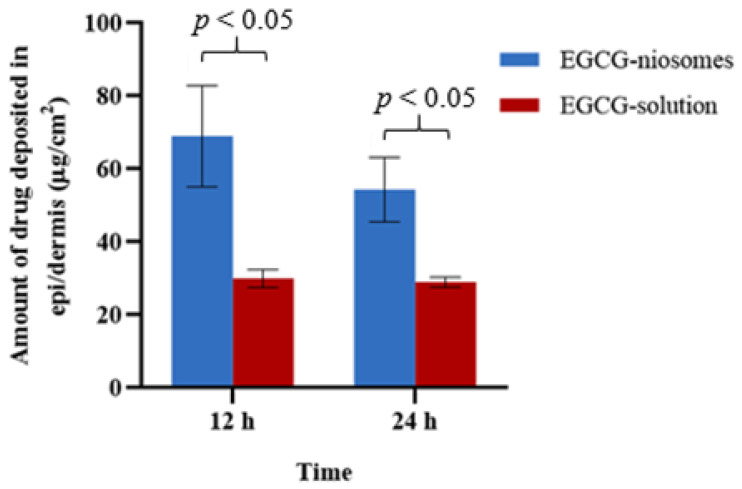
The amount of drug deposited in the human skin layers from EGCG-niosomes and EGCG-solution (mean ± SD, *n* = 3).

No drug was found in the receptor chamber at the end of the permeation study, and this could be caused by hydrolysis of EGCG in the aqueous medium and the limited sensitivity of the HPLC method. Figure 5 shows the amount of EGCG deposited in the human skin from niosomes and EGCG solution at 12 and 24 h. The deposition of EGCG-solution were 30.02 ± 2.45 μg/cm^2^, 29.00 ± 1.36 μg/cm^2^ at 12 h and 24 h, respectively. The drug deposition levels of EGCG-niosome were 69.0 ± 13.87 µmg/cm^2^ and 54.38 ± 8.86 µmg/cm^2^ at 12 h and 24 h, respectively. When the deposition of the EGCG-niosome and the drug solution was compared at 12 and 24 h, it was discovered that the deposition from the EGCG-niosome was about 2-fold higher than that of the drug solution.

### 3.10. Visualisation of Skin Penetration and Deposition

**Figure 6:**
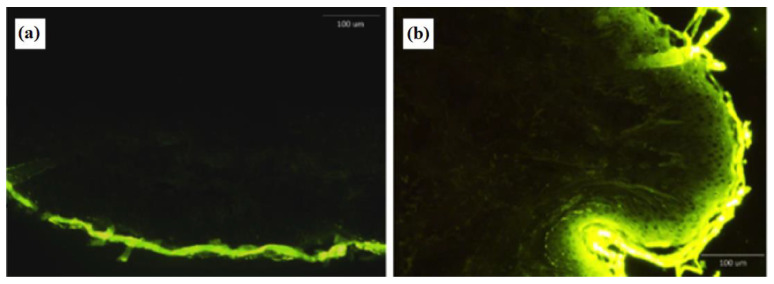
Sections of the full-thickness human skin after been treated with Fluorescein 5(6)-isothiocyanate (FITC) solution (**a**) and FITC-loaded niosomes (**b**) after 12 h.

A small amount of fluorescence was seen in the epidermis after 12 h of ethanol solution application (Figure 6). On the other hand, the niosome carrier improved fluorescence penetration through the SC and greater fluorescence intensity can be observed in the epidermis and dermis. This result matched with the results obtained from the deposition studies and confirmed that niosome could increase drug deposition into the human skin layers.

### 3.11. The Pharmacological Effects of EGCG-Niosomes on Fbs

#### 3.11.1. Cell Viability after UVA-Irradiation

**Figure 7:**
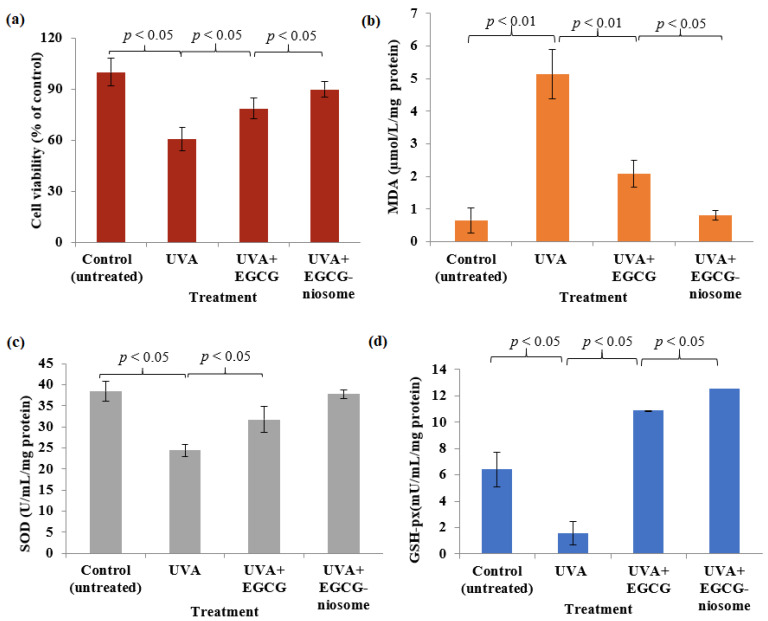
(**a**) Cellular viability after UVA-irradiation and treatment with EGCG and EGCG-niosomes. The effect of EGCG and EGCG niosomes on (**b**) intracellular malondialdehyde (MDA) level (**b**), (**c**) superoxide dismutase (SOD) and (**d**) glutathione peroxidase (GSH)-px after UVA-irradiation (mean ± SD, *n* = 3).

UVA-irradiation caused substantial reduction in cell viability of 40% when compared to control (*p* < 0.05) (Figure 7a). Fbs treated with EGCG-niosomes demonstrated higher viability, (*p* < 0.05) as compared to the UVA-irradiation group, considerably greater than the group treated with EGCG (*p* < 0.05).

#### 3.11.2. Intracellular MDA Level and the Antioxidant Enzyme Activities

The extent of cellular lipid peroxidation can be determined by measuring intracellular MDA levels. As shown in Figure 7b, intracellular MDA levels after UVA-irradiation was 5.12 ± 0.76 µmol/L/mg protein, which was significantly higher compared to untreated cells (*p* < 0.01), showing that UVA has a strong oxidative effect on skin cells. The intracellular MDA levels of Fbs treated with EGCG-niosomes were much lower (0.80 ± 0.33 µmol/L/mg protein) compared to Fbs treated with free EGCG (2.08 ± 0.33 mol/L/mg protein). Figure 7c,d shows that the activity of the intracellular antioxidant enzymes following UVA-irradiation were reduced significantly for both SOD and GSH-px. EGCG-niosome had a greater enhancing effect on the SOD activity compared to the pure drug, but the difference was insignificant (*p* > 0.05) (Figure 7c), the level of SOD was 36.48 ± 1.98 µ/L/mg protein, the group treated with free EGCG was 31.92 ± 1.67 µ/L/mg protein. The GSH-px level in Fbs after UVA irradiated was increased by EGCG-niosomes to 12.53 ± 0.01 mU/L/mg protein, significantly higher when compared to the group treated with free EGCG (10.88 ± 0.55 mU/L/mg protein) (*p* < 0.05) (Figure 7d).

### 3.12. Cellular Uptake of Niosome by Fbs Cells

**Figure 8:**
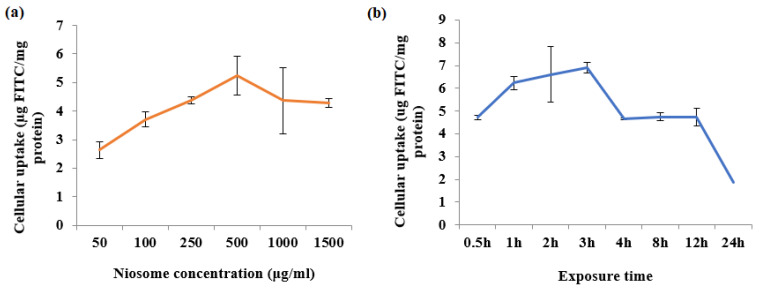
(**a**) Effects of niosome concentrations and (**b**) duration of exposure on the uptake of vesicles by Fbs.

Three factors influenced cellular uptake including niosome concentration, exposure duration, and incubation temperature were studied. Figure 8a shows that increasing the concentration from 50 to 500 µg/mL enhanced cellular uptake, but a further increase from 500 µg/mL did not lead to further increase. Figure 8b shows that cellular uptake was time dependent. Maximum uptake was reached after 3 h before it was declining. At 37 °C, cellular absorption was 6.89 µg FITC/mg protein, 6-fold higher than at 4 °C (0.90 µg FITC/mg protein), which indicated that this process requires energy. No cellular uptake was observed in cells incubated free FITC and no intercellular fluorescence was detected. Confocal microscopy was used to examine whether niosomes were taken up into the cells; it provides the observation of a three-dimensional cross-sectional images of the cells and the location of niosomes within the cells. Labelling the cells with CellTracker and DAPI allowed the cells to be visible under the microscope.

**Figure 9:**
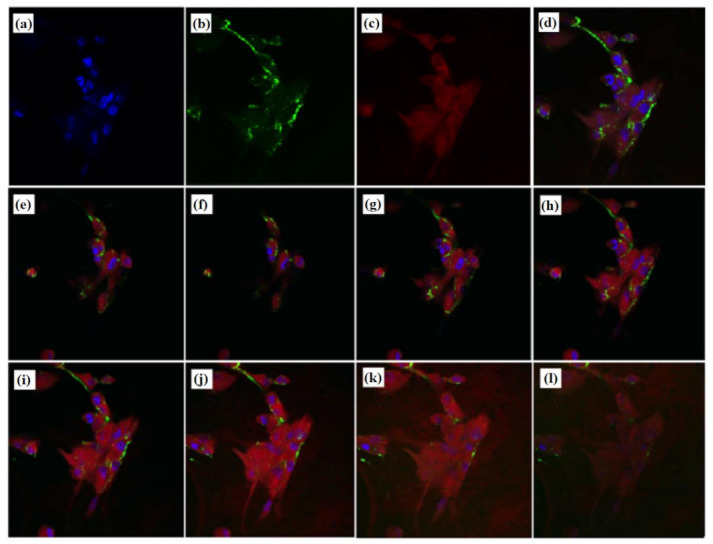
Confocal laser scanning microscopy images of Fbs after incubation with FITC-labelled niosomes for 2 h at 37 °C showing perinuclear accumulation of particles. Nuclei: blue (**a**), FITC-labelled niosomes: green (**b**), cytoplasm: red (**c**), merged images (**d**) confirming uptake of intake niosomes. Eight images of optical sections taken in the vertical axis at interval of 1 µm from the apical surface (**e**–**l**) from left to right; top to bottom, depths 0, 1, 2, 3, 4, 5, 6 and 7 μm, demonstrating particle internalisation. Magnification (600×).

Figure 9a,b illustrates the distribution of green FITC-labelled niosomes inside Fbs after 2 h of uptake. The images indicated that the niosomes were distributed throughout the cytoplasm and perinuclear region. The planar section observation confirmed that FITC was internalised rather than just adsorbing on the cell membranes. 

## 4. Discussion

In this study, EGCG-loaded niosomes were fabricated and optimised by using first a 2^6−2^ fractional factorial design followed by a central composite design. The development of niosomes involves many factors, which may affect their properties such as size and encapsulation of the drug in niosomes. The traditional experimental approach implies altering one factor at a time while keeping the other constant. In this case, to evaluate a certain number of factors, a great effort and long period of time are required. In contrast to the traditional method, utilisation of fractional factorial design is able to provide the maximum amount of information with the least experiments [38]. From a pharmaceutical viewpoint, EE is one of the most important attributes of niosome formulation; a high EE would result in less time and effort spent removing unentrapped material and a greater therapeutic effect of the product [39].

The effect of drug content used in preparation on EE% was statistically significant. Generally, increasing drug amount led to improved EE, but in the EGCG-niosomes, further increase in the drug amount above 1.4 mg showed a decrease of EE. This might be due to saturation of drug entrapment, i.e., further addition of the drug was not able to induce more drug entrapped. The ratio of CH to surfactant was found to significantly influence the entrapment of the EGCG-niosome. CH acts as a membrane stabiliser. It increases rigidity of the bilayer and reduces leakage of drugs from the vesicles; it has been reported that as the amount of CH increases in the formulation, the entrapment efficiency of the drug also increases [40]. Nevertheless, the addition of CH above a certain level may cause disruption of the regular vesicle structure, thus decreasing the entrapment [41]. This finding is consistent with those reported by other researchers. Incorporation of CH into Span 60 niosome loaded with flurbiprofen resulted in an increase of EE from 55% to 67%, but EE was reduced by 30% when CH was increased to 60% [42]. The EE of caffeine decreased from 80% to 50% when the molar ratio of CH to surfactant increased from 3:7 to 3:5 [43]. In the current CCD study, it was obvious that the response surface had curvature in the optimisation phase of both niosome formulations. It indicated that in both niosome preparation, as the CH amount in preparation increased, the EE increased at first, whereas after a certain level, further increase of CH caused a decrease of EE.

The optimised nano-size EGCG-niosomes had an average particle size of 235.4 ± 15.64 nm and a zeta potential of −45.2 ± 0.03 mV. SEM confirmed the findings obtained from Zetasizer, that niosomes were in the 200 to 300 nm size range with a narrow distribution. In topical drug delivery, the particle size of the carriers plays an important role in penetration across the skin barrier. Studies have shown that when the particle size of carriers is greater than 600 nm, no skin deposition was observed. Carriers with a smaller particle size, such as 300 nm promote dermal delivery, while a size lower than 300 nm may result in excessive transdermal drug transport [17]. The zeta potential is an extremely useful measure of a formulation’s stability. A zeta potential of less than −30 mV indicates high stability [44]. Adding DCP in the EGC-niosomes resulted in a much lower zeta potential than −30 mV. 

The EGCG-niosomes achieved a high EE of 53.05 ± 4.46%, and DSC and FTIR showed that EGCG was successfully encapsulated in the niosomes. In EGCG-niosomes, an additional peak was observed between 100 to 150 °C, indicating a surfactant-cholesterol interaction. This interaction is crucial, as CH acts as a membrane stabiliser in niosomes. Drug release from EGCG-niosomes showed a biphasic pattern, where an initial burst release and a subsequent slow release were observed. The release kinetics followed the Korsmeyer release model, (*r*^2^ = 0.996), demonstrating an anomalous diffusion mechanism regulated by many processes [45,46]. When it comes to topical drug delivery, this type of release pattern is appealing because the initial fast release improves drug penetration, while the subsequent sustained release provides the drug delivery over a longer period to maintain a therapeutic level in the skin and reduces the frequency of reapplication [47,48,49].

According to research, the use of the niosome carrier has a considerable impact on enhancing topical drug penetration, as well as increasing drug deposition in the human skin, which are both advantageous for dermal formulations. As such, niosomes have been extensively used in topical treatments [17,21,28,50,51]. A number of theories have been put forward to explain their ability to enhance penetration, firstly the adsorption and fusion of carriers onto the skin’s surface results in a significant thermodynamic activity gradient of the drug at the surface of the carriers and the skin’s surface, which serves as a driving force for drug penetration into the skin [52,53,54]. Secondly, disruption of the tightly packed lipids that occupy the extracellular spaces of the SC increases drug permeability through structural alteration of the SC. Thirdly, the carrier may disturb the densely packed lipids of the SC to promote drug penetration by modifying the SC structure. Lastly nonionic surfactants may act as penetration enhancers, increasing membrane fluidity [26,32,52,55]. Finally, niosomes alter the SC characteristics by reducing trans-epidermal water loss, increasing SC hydration and leading to the relaxation of its tightly packed cellular structure and, hence, better penetration [52,53]. Ethanol is also known as a penetration enhancer [56]. It reduces the phase transition temperature of SC lipids, improving fluidity of SC. In addition, ethanol imparts soft properties to the carrier’s membrane, facilitating vesicle skin penetration [17]. On the other hand, no drug was detected in the receptor chamber of the Franz diffusion cells, indicating that EGCG did not permeate across the skin. The entrapped EGCG and released EGCG molecules may partition into and diffuse through the SC. A drug depot may be formed in the SC, and then the remaining free drug and vesicles penetrate farther into the epidermis until they reach the interface between the SC and the epidermis. The free drug, as well as any remaining intact vesicles, are subsequently released into the skin layers. It is possible that the drug was metabolised by the enzymes in the skin. Catechin is unstable in aqueous environments, and it has been shown that it is rapidly hydrolyzed or degraded. Based on these findings, it may be feasible to explain why no drug was detected in the receptor chamber.

The assay is based on the ability of the dye sulforhodamine B to bind electrostatically and pH-dependently on protein basic amino acid residues of TCA fixed cells. A significant reduction in cellular viability was observed after UVA-irradiation; however, the Fbs viability was significantly improved by both EGCG solution and EGCG-niosomes, with the EGCG-niosomes showing greater protective effects against UVA-irradiation. ROS may cause cell and tissue dysfunction, which is partly manifested as lipid peroxidation. Malondialdehyde (MDA) is the main product of lipid peroxidation, and it reveals the level of cell damage under oxidation [57]. In addition, antioxidant molecules in the skin interact with ROS or their by-products such as MDA to minimise the deleterious oxidation effect. After being exposed to oxidative stress, the antioxidants in the skin SOD and GSH-px are activated [58]. UV irradiation causes an accumulation of ROS in the skin, overwhelming the tissue antioxidants, and thus it causes oxidative stress-related skin problems [6]. ROS may be alleviated by SOD and GSH-px. The decrease in SOD and GSH-px levels observed after UV irradiation might be attributed to the formation of a large number of free radicals that exceeded the antioxidant enzymes’ scavenging capacity [59]. Furthermore, the reduction in enzymatic activity might be related to enzyme inactivation caused by ROS damage to DNA. MDA content, which indicates the lipid peroxidation state, increases following UV irradiation, showing damage induced by oxidative stress. EGCG is a polyphenol compound with a wide range of pharmacological actions. This compound has strong antioxidant properties. It is capable of scavenging ROS or their precursors, blocking ROS synthesis and upregulating antioxidant enzymes [60]. Following UV irradiation, skin Fbs treated with EGCG-niosomes had higher SOD and GSH-px activity compared to UV-treated cells. Furthermore, the MDA levels in the EGCG solution-treated group were lower than in the UV group. EGCG-niosomes showed significantly higher antioxidant activity, which might be due to the following explanations: when in cell culture, the medication is subject to auto-oxidation, but within a vesicle, it is somewhat shielded from destruction [61]. Furthermore, the drug-loaded niosomes produced prolonged release, keeping the level of the drug constant, resulting in an increased antioxidant effect. Furthermore, the carrier may influence drug internalisation by cells [62]. The improved antioxidant activity of EGCG encapsulated in the niosome carrier prompted researchers to investigate niosome-cell interactions. 

Since free FITC had difficulties penetrating cells, the increased FITC intake should be attributed to the niosome carriers. Many studies have indicated greater drug absorption mediated by drug carriers; tamoxifen citrate loaded niosomes showed in an in vitro study on MCF-7 breast cancer cells that the amount of cellular uptake and cytotoxicity of tamoxifen were greatly enhanced when it was loaded in niosomes. Incorporating antimicrobial agents into carrier systems, such as nanoparticles or microemulsions, might be a successful technique for increasing cellular uptake [62]. Furthermore, niosomes loaded with salidroside improved the drug’s intracellular absorption by both human epidermal immortal keratinocytes and human embryonic skin fibroblasts [53]. 

Endocytosis is a primary mechanism through which cells internalise chemicals and macromolecules. It is essential for cell-to-cell communication and cell-to-microenvironment communication [63]. To internalise foreign particles, human cells employ multiple endocytosis processes. Phagocytosis, macropinocytosis, clathrin-mediated endocytosis, and caveolae-mediated endocytosis are all examples of endocytic processes [64,65,66]. Endocytosis demands energy, as opposed to passive transport, which does not involve any expenditure of energy [64]. According to a study, depending on their size, liposomes are mostly endocytosed by clathrin- or caveolae-mediated endocytosis [65]. Recent findings have revealed that endocytosis of niosomes is an energy-dependent process, follows a concentration- and time-dependent pattern and has a saturation point [66]. As a result, it is likely that cell surface proteins are involved in the process of niosome endocytosis. Niosome carriers have the potential to increase cellular absorption of encapsulated compounds, even if the medication has a low permeability into the cells. Further studies are required to fully understand the uptake process.

## 5. Conclusions

In this work, the niosome-carrier system was fabricated to encapsulate EGCG for cutaneous administration. Based on the findings, we can conclude that EGCG-niosomes can penetrate the skin barrier and improve drug deposition in the viable layers. Because of increased cellular absorption and based on the studies of the antioxidant enzymes, EGCG-niosomes demonstrated an improved antioxidant effect on skin cells. Because antioxidants have numerous roles in skin health, this topical formulation has the potential to be used in the treatment of skin diseases. Furthermore, in both the pharmaceutical and cosmetic industries, this carrier has the potential to be used as a dermal drug carrier for a variety of bioactive compounds.

## Figures and Tables

**Table 1:** 2^(6−2)^ screening design, providing values and coded units with centre points.

Factors	Factor Setting		
	Low (−1)	Centre (0)	High (+1)
Surfactant type (X_1_)	Tween 40	nil	Span 60
Drug amount (mg) (X_2_)	1	5.5	10
Molar ratio of CH to surfactant (X_3_)	1:4	7:8	3:2
DCP content (µmol) (X_4_)	2	6	10
Hydration medium amount (mL) (X_6_)	10	17.5	25
Hydration time (min) (X_6_)	30	75	120

**Table 2:** Optimisation of epigallocatechin gallate (EGCG)-niosome by central composite design.

Factors	Factor Setting				
	−1.4	−1	Centre (0)	+1	+1.4
Drug amount (mg) (X_1_)	0.58	1	2	3	3.4
Molar ratio of CH to surfactant (X_2_)	3:1	1:2	1:1	3:2	17:10

**Table 3:** Screening design of EGCG-niosome showing variables in coded values and response EE (%).

Run Number	Variables						Response (Y); EE (%)
	X_1_	X_2_	X_3_	X_4_	X_5_	X_6_	
1	1	1	−1	−1	1	1	2.3
2	1	1	1	1	1	1	47.9
3	1	1	1	−1	1	−1	24.5
4	−1	1	1	1	−1	1	5.7
5	−1	1	1	−1	−1	−1	12.6
6	−1	1	−1	1	1	−1	27.8
7	1	−1	1	−1	−1	1	49.0
8	1	−1	−1	1	1	1	15.8
9	1	−1	1	1	−1	−1	45.8
10	−1	0	0	0	0	0	18.3
11	−1	−1	1	1	1	1	22.7
12	−1	−1	−1	1	−1	1	17.0
13	1	1	−1	−1	−1	1	30.0
14	1	0	0	0	0	0	42.7
15	−1	0	0	0	0	0	24.6
16	−1	−1	−1	−1	1	1	46
17	1	−1	−1	−1	1	−1	48.7
18	−1	−1	−1	−1	−1	−1	21
19	1	1	−1	1	−1	−1	38.7
20	1	0	0	0	0	0	24.8

**Table 4:** Main effects of single factors and two-factor interactions in EGCG-niosome variable screening.

Factor	Response (EE%)	
	Standardized Effect	Contribution (%)
X_1_-Surfactant	15.58	28.64
X_2_-Drug amount	−6.98	5.74
X_3_-Molar ratio of CH to surfactant	−9.20	9.99
X_4_-DCP content	−1.00	0.12
X_5_-Hydration amount	−0.06	0.04
X_6_-Hydration time	−0.93	0.01
[X_1_ X_2_] = X_1_ X_2_ + X_3_ X_5_	2.60	0.80
[X_1 ×_ 3] = X_1_ X_3_ + X_2_ X_5_	−0.52	0.03
[X_1_X_4_] = X_1_ X_4_ + X_5_X_6_	1.82	0.39
[X_1_X_5_] = X_1_ X_5_ + X_2_ X_3_ + X_4_ X_6_	−6.22	4.57
[X_1_X_6_] = X_1_X_6_ + X_4_ X_5_	−2.65	0.83
[X_2_X_4_] = X_2_ X_4_ + X_3_ X_6_	6.48	4.83
[X_2_X_6_] = X_2_ X_6_ + X_3_ X_4_	1.50	0.27

**Table 5:** Summary of analysis of variance (ANOVA) for the 2^(6−2)^ factorial design for EGCG-niosome variable screening.

Source	Sum of Squares	Df **	Mean Square	F Value	*p*-Value
Model	4566.98	13	352.25	9.12	0.023
X_1_-Surfactant	32.49	1	32.49	0.84	0.411
X_2_-Drug amount	2213.7	1	2213.7	57.32	0.002 *
X_3_-Molar ratio of CH to surfactant	566.40	1	566.44	14.67	0.019 *
X_4_-DCP content	257.60	1	257.60	6.67	0.061
X_5_-Hydration amount	277.22	1	277.22	7.18	0.055
X_6_-Hydration time	707.56	1	707.56	18.32	0.013 *
[X_1_ X_2_] = X_1_ X_2_+ X_3_ X_5_	31.56	1	31.36	0.81	0.419
[X_1_X_3_] = X_1_ X_3_+ X_2_ X_5_	58.52	1	58.52	1.52	0.286
[X_1_X_4_] = X_1_ X_4_+ X_5_X_6_	102.01	1	102.01	2.64	0.179
[X_1_X_5_] = X_1_ X_5_+ X_2_ X_3_+ X_4_ X_6_	72.25	1	72.25	1.87	0.243
[X_1_X_6_] = X_1_X_6_+ X_4_ X_5_	238.70	1	238.70	6.18	0.068
[X_2_X_4_] = X_2_ X_4_+ X_3_ X_6_	12.60	1	12.60	0.33	0.598
[X_2_X_6_] = X_2_ X_6_+ X_3_ X_4_	13.69	1	13.69	0.35	0.584
Lack of fit	21.84	2	10.92	0.16	0.859
Pure Error	123.73	2	61.86		
Cor Total ***	5784.34	19			

* statistically significant *p* < 0.05, R-Squared = 0.96, Adj R-Squared = 0.861, Pred R-Squared = 0.598. ** df: degree of freedom. *** Cor Total: corrected total sum of square.

**Table 6:** Optimisation design of EGCG-niosome showing variables in coded values and responses.

Run Number	Type	X_2_	X_3_	Response Entrapment Efficiency (%)
1	Center	0	0	49.1
2	Axial	0	−1.4	47.5
3	Fractional	−1	+1	11.6
4	Center	0	0	54
5	Center	0	0	39.1
6	Axial	0	+1.4	7.3
7	Fractional	−1	−1	43.6
8	Axial	+1.4	0	12.1
9	Center	0	0	46
10	Fractional	+1	−1	34.6
11	Fractional	+1	+1	12.8
12	Axial	−1.4	0	30.5
13	Center	0	0	47.1

**Table 7:** Analysis of Variance (ANOVA) of the drug entrapment efficiency.

Source	Sum of Squares	Df **	Mean Square	F Value	*p*-Value
Model	3232.04	5	646.81	27.8	0.0002
X_2_-drug amount	1530.47	1	1530.47	65.78	0.0001 *
X_3_-Molar ratio of CH to surfactant	142.99	1	142.99	6.15	0.0423 *
X_2_X_3_	26.01	1	26.01	1.12	0.3255
X_2_^2^	1096.54	1	1096.54	47.13	0.0002
X_3_^2^	628.49	1	628.49	27.01	0.0013
Residual	162.86	7	23.27		
Lack of fit	46.05	3	15.35	0.53	0.6879
Pure Error	116.81	4	29.20		
Cor Total ***	3396.90	12			

* statistically significant *p* < 0.05; ** df: degree of freedom; *** Cor Total: corrected total sum of square.

**Table 8:** Optimised formulation composition for EGCG-niosome.

Formulation Variables	EGCG-Niosome
Surfactant (X_1_)	Span 60
Drug amount (mg) (X_2_)	1.4
Molar ratio of CH to surfactant (X_3_)	0.9
DCP amount (µmol) (X_4_)	2
Hydration medium volume (mL) (X_5_)	10
Hydration time (h) (X_6_)	2
EE (%)	53.05 ± 4.46

**Table 9:** Drug release kinetic parameters of EGCG niosomes.

**Formulation**	Korsmeyer-Peppas Model			Higuchi Model		First-Order		Zero-Order	
	$r^{2}$	n	$k_{k}$	$r^{2}$	$k_{h}$	$r^{2}$	$k_{1}$	$r^{2}$	$k_{0}$
**EGCG-niosomes**	0.996	0.461	3.885	0.876	2.555	0.832	0.002	0.521	0.077

## Data Availability

The data presented in this study are available on request from the corresponding author.
